# Supplementary material for: Vector competence for Oropouche virus: A systematic review of pre-2024 experiments
Source: PLoS Negl Trop Dis. 2025 Apr 30;19(4):e0013014. doi: 10.1371/journal.pntd.0013014 (PMC12043118; doi:10.1371/journal.pntd.0013014)
Supplement: Attachment — Submitted filename: Response to reviewers.docx [file pntd.0013014.s004.docx]

We thank the reviewers for their thoughtful reading of, and comments on our manuscript. We have provided a point-by-point response to their comments below.

**Reviewer #1:**

**Key Review Criteria Required for Acceptance?**

**Methods**
Were other search terms considered, such as simply “Oropouche” or “OROV” or other spellings?

*Only “Oropouche” was considered, we have revised the text to make this clear (line 84-85).*

Needing raw data available could have been a limitation – was this exclusion criteria applied to any potential publication?

*No papers were identified that did not have raw data available, we added this information to line 90.*

Pg 4: some of the results are repeated here and should be removed (i.e., number of publications found by method). In addition, citation searching was mentioned but not described – were only included publication citations searched?

*We have added information on citation searching to lines 91-92.*

Pg 4, 98: PubMed

*Spelling error has been revised as requested (line 108).*

**Results**

No concerns

**Conclusions**

No concerns

**Editorial and Data Presentation Modifications?**
(No Response)

**Summary and General Comments**

This manuscript succinctly describes a highly important systematic review to determine the existence of vector competency studies for Oropouche virus. The analysis and interpretation are very good, and the figures are excellent.

*We thank the reviewer for their support of our paper.*

**Reviewer #2:**

**Key Review Criteria Required for Acceptance?**

**Methods**
PubMed was the only database used.

*PubMed was the only database used, we have revised the text to make this clear (line 84).*

Methodology for citation searching is not clear.

*We have added information on citation searching to lines 91-92.*

The term PRISMA is used in figures and supplemental files only.

*We have added a sentence to the results defining the PRISMA reporting guidelines we used (lines 106-107).*

Reference [1] is a preprint and should not be used as a basis for discussion of changes in clinical presentation.

*This preprint has since been published in a peer-reviewed journal (Lancet Infect Dis), and we have updated the citation (line 42).*

Comment on recent genetic analyses (Lines 38-40) is based on study by Naveca et al in nature medicine (2025, "Human outbreaks of a novel reassortant Oropouche virus in the Brazilian Amazon region") and needs to be cited.

*Revised as requested (line 40).*

**Results**

Figures mentioning PRISMA without context in the text.

*PRISMA is now discussed on lines 106-107 of the results.*

Figure 2a: visualization of publication dates is not clearly labeled

*We have added the x-axis title of “year”, and relabeled the y-axis to “vector competence publication” (Figure 2a).*

Figure 4: Colors are not defined in legend. Usefulness of this figure is unclear.

*We added a legend to figure 4b showing that the different colors represent the different vector species (same color-coding throughout Figures 2-5).*

Figure 4c: Use of OROV titers is unclear.

*This graph is intended to show the broad range (~5 logs) of titers that have been tested to determine vector competence for OROV. We have added text to line 129, to make the importance of this clearer.*

Figure 5a: Experiments where virus concentration was unknown are plotted at far right of the x-axis: The legend overlays this portion of the graph and the number of points at "?" are unclear.

*We have shifted the legend so it does not overlap the graph (no data points were covered), and added to the legend the number of data points per species (lines 261-262).*

Figure 6: This study is so different from the others that its inclusion is questionable. Transmission study was complicated and unclear whether its methodology was appropriate, so viability of the conclusion "No OROV transmission" is in question. Additionally, I needed to combine information from both this figure and lines 140-142 of the text to understand the study methodology utilizing the 2-day old mice. This should have been more clear in the text itself.

*We have revised the text to make these uncommon methods more clear (lines 153-155) and added explanation of why these results were included (lines 147-150).*

Line 51: Reference [7] utilizes more methodology than Culicoides abundance to incriminate midges in transmission.

*While we state that Culicoides are in high abundance, we also note that OROV has been isolated from C. paraensis multiple times during the 1975 outbreak in Brazil, and these together contribute to their incrimination as the principle vector (lines 49-51).*

Lines 47-63: This paragraph needs to contain more data on Culicoides because they have been shown to transmit.

*Revised as requested (lines 52-54).*

Line 60: conclusion about low OROV prevalence in humans due to low detection rates in mosquitoes is flawed. When these urban vectors were collected in relation to human viremia, and low vector competence more reasonable.

*We have removed the statement regarding low prevalence in humans (line 62-63).*

Line 62: Where has speculation about potential vector shift been published?

*We have added references and included the note that no Culicoides species have been reported in Cuba where OROV is now endemic (lines 66-67).*

Line 66: Please briefly describe the components of the data standard and how you used them.

*Revised as requested (line 70-72).*

Line 119: "range of OROV quantified" needs better clarification

*Revised as requested (line 129).*

Lines 140-142: Better communication about this study is needed, as reasoning and sequence of mosquito feeding, baby mouse blending, and IC injection is confusing. It is also worth noting the potential weaknesses of this paper.

*We have added more details to make the methods of this paper clearer (lines 153-155), and discussed our reasoning for including it on lines 147-150.*

**Conclusions**

Line 158: vector instead of host

*Revised as requested (line 172).*

Line 158: conclusion that initial infection of midgut cells blocked OROV infection is not supported by data presented in this review. If retained, reference is needed here and presentation of methodology from this reference is needed in the review manuscript.

*We have revised this section to further clarify (lines 171-172).*

Lines 165-166: "Culicoides midges are highly competent..." and "few studies evaluating...competence" are conflicting statements. Suggest re-wording for clarity of intention.

*We have revised to note there are few studies demonstrating their competence (line 180).*

Lines 169-170: Review content suggests that Culicoides are already known to actively transmit OROV. "...could someday pose a risk" is not supported and one wonders if this statement was meant for Culex.

*We have clarified this sentence to highlight it is unknown the role of Culicoides in the current outbreak, or future outbreaks in new locations (lines 191-192).*

Conclusion discusses "shift in vector-virus interactions" (Line 181), which is appropriate. However, Introduction discusses changes in clinical presentation of Oropouche fever (Lines 42-43) which has not been supported. Furthermore, this statement on lines 42-43 is based on a preprint, which should not be used to guide clinical work.

*We have revised this to state that the new reassortant “may also result in changes in clinical presentation (line 43). As noted above, reference #1 has since been published in a peer-reviewed journal (Lancet Infect Dis), we have updated our citation to reflect this.*

**Editorial and Data Presentation Modifications?**
Abstract Lines 17-18: "risk of establishment in regions with frequent travel to..." This needs to be re-phrased to allow full thought processes about travel, vector ranges, human infections, animal infections, and spread of pathogens via human travel. At this time, it makes a lot of assumptions.

*We appreciate this suggestion, and have revised this to read "and whether local vectors and wildlife communities create any risk of establishment in non-endemic regions” (lines 17-18).*

Summary Line 21: Caution in using the term "major threat" without quantifying current threat or prognosticating future continued threat.

*We have revised to “an urgent threat” (line 21).*

**Summary and General Comments**

More text discussion is needed about Culicoides as a vector, since this is currently determined to be the major vector of OROV.

*Revised as requested (lines 52-54).*

Overall this review is well done and informative. Needs some refining.

*We thank the reviewer for their support and their comments which have strengthened and clarified multiple sections of the paper.*

**Reviewer #3:**

**Key Review Criteria Required for Acceptance?**

**Methods**

The study methods are well designed and address the specific research objectives: to review vector competence studies for Oropouche virus.

**Results**

Results and figures are well done.

**Conclusions**

Conclusions are clear and summarise the work.

**Editorial and Data Presentation Modifications?**
Data presentation is excellent, clear and easy to understand. The authors have done well to visually display the different experimental conditions used in each study and the potential impact this would have on the outcomes.

**Summary and General Comments**

This is a very well written paper that synthesizes vector competence studies. The authors have done very well summarising the different experimental methods and vector characteristics across studies which can impact the results and comparisons between experimental studies.

As one might expect, the key findings are that we don't know much about which species are potential vectors for Oropouche virus and that much more work is needed in this space. However, because this is a review of all the available studies it would be more impactful to include more information about the vectors that were experimentally competent. Many of the results and discussion were comparing the higher competence in Culicoides to mosquitoes, but aside from an introductory paragraph, I would have liked to know more about about the Culicoides species themselves that did demonstrate competence. It would be good to discuss and compare how those species differ from each other in the experimental studies, and include any other information that may be important for their vectorial capacity such as difference in their distributions, or known seasonality that might be important in the context of Oropouche? Are both of these species known to exist in Cuba, or other places that have historic or current epidemics?

*We have added additional discussion about Culicoides, their transmission and known distribution to lines 185-190.*

Similarly I was wondering if there is any known evidence on non-human hosts of Oropouche, and if so, it would be good to include a few sentences. It is interesting to note that in L61 you suggest that many blood-feeding arthropods are infected but not all transmit - what could be infecting them with such low transmission in human outside of outbreak years? This is not specific to vector competence, but is important in the context of vectorial capacity, which is in the discussion.

*We have added additional discussion regarding other non-human hosts of OROV and their role maintaining sylvatic OROV (lines 52-54).*

Otherwise this study was excellent and the information is important for designing future studies and interventions for Oropouche virus.

L14: What constitutes as high? Could you include a value from the results as was done for the mosquitoes

*Revised as requested (line 15).*

L59: Is this detection rate in humans or mosquitoes - both are mentioned, suggestion to be more explicit.

*Revised to make clear this is the mosquito virus detection rate (line 62).*

L141: At first this read to me that the mice were immediately blended (which was not what I expected to see in a vector study this morning), suggestion to be explicit that this is referring to the mosquitoes.

*It was surprisingly the mice that were “blended” then injected into naïve mice to detect virus (the paper’s original text included below). We have revised the text to make this more clear (lines 153-155).*

“Two weeks later, the infected mosquitoes were allowed to bite 2-day-old mice; the mice were then immediately blended in 5 to 10 cc diluent and inoculated into infant mice i.c. in an attempt to recover virus.”

L158: remove the brackets at and add another sentence here about why IT injections are not representative of natural infection and transmission risk.

*Revised as requested (lines 174-176).*

L178: Could you include an example? Ae. albopictus and dengue would be a relevant one.

*Revised as requested (lines 202-203).*
