# Supplementary material for: Vector competence for Oropouche virus: A systematic review of pre-2024 experiments
Source: PLoS Negl Trop Dis. 2025 Apr 30;19(4):e0013014. doi: 10.1371/journal.pntd.0013014 (PMC12043118; doi:10.1371/journal.pntd.0013014)
Supplement: Attachment — Submitted filename: Response to reviewers R2.docx [file pntd.0013014.s005.docx]

We thank the reviewers for their thoughtful reading of, and comments on our manuscript. We have provided a point-by-point response to their comments below.

**Reviewer #1:**

**Key Review Criteria Required for Acceptance?**

**Methods**
My concerns were mostly addressed, but the actual methods for citation searching are still missing. Were the citations of only included articles searched, or were the citations of non-included articles considered? Was it all articles in that category, or were they targeted somehow?

*We have expanded in this section to make more clear how the additional references were identified (lines 91-95).*

**Results**

No more concerns

**Conclusions**

No concerns

**Editorial and Data Presentation Modifications?**
(No Response)

**Summary and General Comments**

(No response)

**Reviewer #2:**

**Key Review Criteria Required for Acceptance?**

**Methods**
Manuscript is much improved and well done. Great job!

Line 22: reads a bit clunky. Since citations are not needed in the Author Summary, recommend simplifying to what you would like to say.

*We revised this sentence to make it less clunky (line 22).*

Lines 113-114 dates appear contradictory.

*We have clarified that there were 2 studies published in 2021 (line 117).*

Lines 146-150 is repetitive with line 117 and can be simplified. e.g., "Anderson et al (1961) evaluated OROV infection and transmission in parentally inoculated Ae. scapularis, Ae. serratus, Cx. quinquefasciatus (referred to as Cx. fatigans in the paper) and Psorophora ferox mosquitoes.

*Revised as requested (lines 120, 149-150).*

Line 154: Please briefly explain what was tested after diluent injection into infant mice

*Revised as requested (line 154).*

Line 170-171: "block of infection" seems a potential overstatement here. Wording "is not" also comes across strong.

*Revised as requested (line 170-171).*

Line 183: Recommend adding the word 'wild', as, "... detection and isolation rates in wild C. paraensis..."

*Revised as requested (line 183).*

**Results**

Reviewer #2: Figure 3: Hexagons appear on the figure between years 1980-1985; but caption states hexagons show data without a collection year

*We have revised the legend to make more clear that the shape (circle/hexagon) refers to the publication year, and the line refers to the year of vector collection (lines 243-245).*

Figure 5: Legend should apply to all graphs in this figure, not just a.

Number of pos samples out of total == Percent of animals tested

*Revised as requested (lines 256-268).*

**Conclusions**

Good

**Editorial and Data Presentation Modifications?**
Accept

**Summary and General Comments**

(No response)

**Reviewer #3:**

**Key Review Criteria Required for Acceptance?**

**Methods**
(No response)

**Results**

(No response)

**Conclusions**

(No response)

**Editorial and Data Presentation Modifications?**
(No Response)

**Summary and General Comments**

(No response)
